# Supplementation of 18β-glycyrrhetinic acid attenuates D-galactose-induced oxidative stress and inflammatory responses in kidneys of weaned piglet

**DOI:** 10.1093/jas/skaf240

**Published:** 2025-07-31

**Authors:** Ruitong Li, Cui Ma, Fuxi Wang, Yaqing Liu, Xinru Wang, Junmin Zhang, Tieying Zhang, Wei Si

**Affiliations:** State Key Laboratory of Animal Nutrition and Feeding, Institute of Animal Sciences, Chinese Academy of Agricultural Sciences, Beijing 100193, China; State Key Laboratory of Animal Nutrition and Feeding, Institute of Animal Sciences, Chinese Academy of Agricultural Sciences, Beijing 100193, China; College of Animal Science, Shanxi Agricultural University, TaiGu 030801, China; College of Animal Science and Technology, Qingdao Agricultural University, Qingdao 266109, China; College of Animal Science and Technology, Qingdao Agricultural University, Qingdao 266109, China; State Key Laboratory of Animal Nutrition and Feeding, Institute of Animal Sciences, Chinese Academy of Agricultural Sciences, Beijing 100193, China; State Key Laboratory of Animal Nutrition and Feeding, Institute of Animal Sciences, Chinese Academy of Agricultural Sciences, Beijing 100193, China; State Key Laboratory of Animal Nutrition and Feeding, Institute of Animal Sciences, Chinese Academy of Agricultural Sciences, Beijing 100193, China

**Keywords:** 18β-glycyrrhetinic acid, D-galactose, kidney, oxidative stress, renal fibrosis

## Abstract

Oxidative stress is a common issue in intensive pig production, threatening kidney health and increasing susceptibility to oxidative damage. 18β-Glycyrrhetinic acid (GA), a pentacyclic triterpenoid derived from the *Glycyrrhiza* genus, exhibits potent antioxidant and anti-inflammatory properties. This study evaluates the potential of GA in mitigating D-galactose (D-gal)-induced renal oxidative injury and explores the underlying mechanisms. Renal oxidative stress was induced in piglets by administering 10 g/kg BW of D-gal for 28 d, followed by a 28-d diet supplemented with 100 mg/kg/d of GA (*n* = 8 per group) to assess its mitigating effects. The results demonstrated that GA supplementation significantly enhanced T-AOC (*P* < 0.05) levels in D-gal-induced piglet kidneys, reduced 8-OHdG levels, increased SOD activity, and upregulated antioxidant genes (*CAT*, *SOD1*, *SOD3*), while downregulating *iNOS* (All *P* < 0.05). GA also reversed the elevated levels of TGF-β (*P* < 0.05) induced by D-gal. Histopathological analysis revealed that GA restored renal structure, reduced inflammation, and alleviated fibrosis. Transcriptomic analysis revealed that GA upregulated antioxidant genes such as *SOD3* and *GSTA1*, while downregulating genes related to inflammation and fibrosis in D-gal-treated piglets. Moreover, GA inhibited the excessive extracellular matrix (ECM)–receptor interactions, PI3K-Akt signaling, and MAPK signaling pathways. Western blot analysis confirmed that GA supplementation significantly reduced PI3K levels (*P* < 0.05), tended to inhibit Akt phosphorylation (*P* = 0.099), and attenuated p38 MAPK phosphorylation (*P* < 0.05). GA also tended to increase Nrf2 expression (*P* = 0.071) and significantly upregulated HO-1 and NQO-1 protein levels (*P* < 0.05). These findings indicate that GA protects against D-gal-induced renal oxidative damage by activating the Nrf2 signaling pathway, while simultaneously alleviating fibrosis and inflammation through modulation of the TGF-β/PI3K/AKT and p38 MAPK pathways.

## Teaser

18β-Glycyrrhetinic acid, a bioactive compound extracted from plants of the *Glycyrrhiza* genus, exhibits potent antioxidant and anti-inflammatory properties, positioning it as a promising candidate for inclusion in animal feed. This study reveals that 18β-glycyrrhetinic acid mitigates renal oxidative stress, inflammation, and fibrosis induced by D-galactose.

## Graphical Abstract

**Graphical Abstract:**
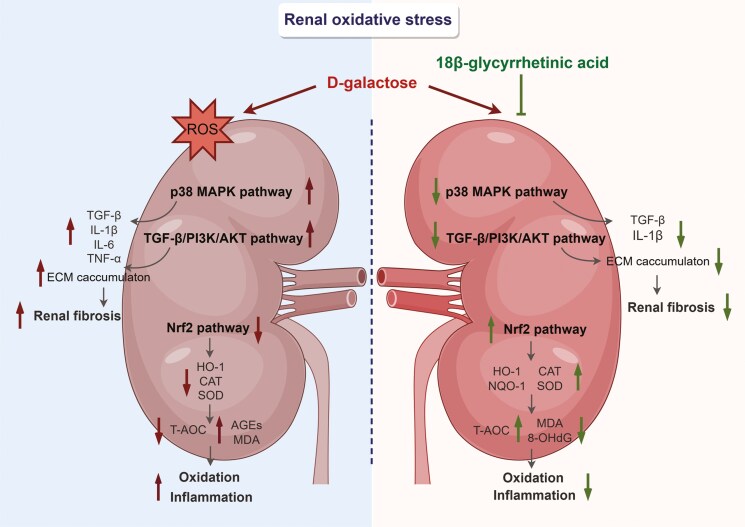

## Introduction

Despite gains in growth rate and feed efficiency, post‑weaning piglet mortality remains high, reaching up to 5.6% during the wean–finish phase (USDA, 2015). Oxidative stress plays a pivotal role in these losses (Zhu et al., 2012). During the weaning-to-finishing transition, piglets experience a surge in reactive oxygen species (ROS) production, leading to tissue damage—particularly in organs such as the liver and gut, which have been the primary focus of previous studies (Buchet et al., 2017; Novais et al., 2020). In addition to these organs, the kidney is particularly susceptible to oxidative injury during this period, due to its rapid postnatal development and high mitochondrial activity, both of which heighten its sensitivity to redox imbalance and oxidative stress (Friis, 1980; Daenen et al., 2019). Previous studies also have shown that renal oxidative stress becomes apparent post-weaning, as reflected by altered glutathione peroxidase (GPX) and superoxide dismutase (SOD) activities, along with dysregulated expression of key redox-related genes, including *GPX3*, *BNIP3*, *MT1*, and *MT2* (Novais et al., 2020, 2021). Oxidative stress is known to trigger renal inflammation and glomerular atrophy in piglets (Marin et al., 2018; Qiu et al., 2022). It also activates the TGF-β signaling pathway, leading to extracellular matrix (ECM) accumulation and fibrosis (Su et al., 2019; Zhang et al., 2023). These pathological changes impair renal function and disrupt physiological homeostasis, causing toxin accumulation that negatively impacts nutrient metabolism and immune responses (Piko et al., 2023). Consequently, growth performance declines, as reflected by reductions in final body weight (BW), average daily gain (ADG), and average daily feed intake, alongside an increased feed to gain ratio (F/G) (Gan et al., 2021). These findings emphasize the importance of focusing on renal oxidative stress in piglets during the weaning stage.

D-galactose (D-gal) has been recognized as an effective agent for modeling chronic oxidative stress and mild inflammation in piglets under conditions that closely mimic commercial production (Li et al., 2020; Han et al., 2021). In weaned piglets, daily oral administration of 10 g/kg BW D‑gal reduces ADG and feed intake while elevating serum MDA, confirming systemic oxidative distress (Han et al., 2021). Although D‑gal-induced renal oxidative injury has not yet been documented in swine, rodent models consistently demonstrate that D‑gal provokes renal oxidative and inflammatory damage. These renal alterations are characterized by heightened AGEs and 8-OHdG levels, impaired key antioxidant enzymes (SOD and GPX), and typical histopathological changes, including tubular edema, vacuolar degeneration, interstitial fibrosis, and inflammatory cell infiltration (Fan et al., 2016; Pan et al., 2021). Taken together, these findings support the D‑gal model as a useful tool to investigate renal oxidative injury in piglets and for evaluating dietary interventions to preserve kidney health in swine husbandry.

Natural plant extracts have shown promise in mitigating oxidative stress and inflammation-related damage (Taïlé et al., 2020; Salehi et al., 2021). One such compound, 18β-glycyrrhetinic acid (GA), is a natural pentacyclic triterpenoid found abundantly in plants of the *Glycyrrhiza* genus (Zhao et al., 2012). GA exhibits a range of biological activities, including antioxidant, anti-inflammatory, and antitumor properties, suggesting its potential for pharmaceutical applications (Li et al., 2024). Research has shown that GA mitigates bisphenol A-induced renal injury by reducing oxidative stress, inflammation, and apoptosis, while enhancing antioxidant enzyme activity and preserving cellular homeostasis (Darendelioglu et al., 2024). Its nephroprotective effects are primarily attributed to the upregulation of nuclear factor erythroid 2–related factor 2 (Nrf2) (Wu et al., 2015; Abd El-Twab et al., 2016). However, the protective effects of dietary GA supplementation on piglet renal oxidative damage remain limited and inconclusive. The present study was therefore designed to test this hypothesis using a D-gal-induced chronic oxidative stress and mild inflammation model, and to evaluate the potential of GA in preventing renal oxidative damage in piglets.

## Materials and Methods

The animal experimental procedures were approved by the Institutional Animal Care and Use Committee of the Institute of Animal Sciences at the Chinese Academy of Agricultural Sciences (approval number IAS 2023-22).

### Animals and experimental design

Twenty-four healthy 28-d-old weaned Large White piglets were procured from Beijing Shunxin Agricultural Co., Ltd (Beijjing, China). The piglets had an average initial BW of 7.50 ± 0.10 kg. After a 7-d adaptation period, they were randomly assigned to three treatment groups, each comprising eight replicates with one piglet per replicate: CK group, received a standard diet; gal group, received a standard diet supplemented with D-gal at a dosage of 10 g/kg BW per day; GA + gal group, received the gal group diet further supplemented with GA at a dosage of 100 mg/kg diet per day. The basal diet was formulated to meet or exceed the nutrient requirements for weaned pigs as outlined by the National Research Council (NRC, 2012). Detailed ingredient composition and calculated nutrient levels are presented in Supplementary Table S1. The diet provided 10.58 MJ/kg of NE, 18.92% CP, and 1.41% SID Lys. A single basal diet was fed to all piglets throughout the 28-d experimental period, and the feed was provided in mash form. The 18β-GA was obtained from Gansu Fanzhi Pharmaceutical Co., Ltd. (Gansu, China) with a purity of ≥98% and D-galactose was acquired from Hubei Yuying Biotechnology Co., Ltd. (Hubei, China) with a purity of ≥99%. The dosages of D-gal were determined based on established references (Han et al., 2021). The formal trial lasted 28 d, during which the piglets had ad libitum access to water and feed. Daily observations and recordings were conducted to monitor their health status.

### Sample collection

On day 28 of the experiment, the piglets were weighed and euthanized to collect kidney tissues. The kidney mass was weighed to calculate the kidney index. Subsequently, approximately 1 cm^3^ of kidney tissue was fixed in 4% paraformaldehyde (PFA) for paraffin embedding, while the remaining tissue was rapidly frozen in liquid nitrogen and stored at −80 °C for further analysis.

### Determination of kidney antioxidant enzyme activities and metabolite contents

Approximately 400 mg of frozen kidney tissue was homogenized in PBS (1:9, w/v) and centrifuged at 12,000 rpm for 20 min at 4 °C to remove debris. The supernatant was collected for analysis. Total antioxidant capacity (T-AOC; BC1315), superoxide dismutase (SOD; BC5165), and malondialdehyde (MDA; BC0025) levels were measured using commercial kits from Solarbio Biotech Co., Ltd. (Beijing, China), following the manufacturer’s protocols. Concentrations of Advanced glycation end products (AGEs; YT-77930O2), 8-hydroxy-2’ -deoxyguanosine (8-OHdG; YT-1447O2), transforming growth factor-β (TGF-β; YT-3652502), interleukin 1beta (IL-1β; YT-042202), interleukin 6 (IL-6; YT-041802), and tumor necrosis factor-alpha (TNF-α; YT-038301) in the kidney were assessed using pig-specific ELISA kits obtained from Jiangsu Meimian Industrial Co., Ltd. (Jiangsu, China). The protein content of each sample was determined by the bicinchoninic acid (BCA) protein assay (23227; Thermo Fisher Scientific, United States).

### Histopathology

The kidney tissues were fixed in 4% PFA overnight at 4 °C and embedded in paraffin. Sections (5 μm thick) were stained with hematoxylin and eosin (H&E) (G1121; Solarbio, China) for morphological analysis and Sirius Red (G1472; Solarbio, China) for qualitative assessment of renal fibrosis. A blinded observer assigned semiquantitative scores for kidney injury based on the percentages of tubules showing cellular necrosis, loss of brush border, interstitial edema, vacuolization, and tubule dilatation (0 = none, 1 = 0-20%, 2 = 20% to 50%, 3 = 50% to 70%3, 4 = more than 70%) (Yu et al., 2015). Sirius Red–stained sections were analyzed using ImageJ software to quantify fibrotic areas. At least 10 fields were examined for each tissue sample.

### RNA extraction and real-time quantitative PCR

The experimental procedures for total RNA extraction from kidney tissues, RNA reverse transcription, and cDNA quantification were performed in accordance with prior research with slight adjustments (Song et al., 2023). The 2^−ΔΔCt^ method, with *GADPH* as the housekeeping gene, was employed to determine the relative expression levels of the target genes. Triplicate analyses were conducted for each sample to ensure robustness and reproducibility. The primer pairs utilized in this study are listed in Supplementary Table S2.

### Western blotting analysis

Kidney tissue was lysed in RIPA buffer (HX1862-2; Huaxingbio, China) with protease inhibitor (HX1863; Huaxingbio, China) and phosphatase inhibitor (HM4065; Huaxingbio, China) to extract total protein. Proteins (30 µg per lane) were separated on polyacrylamide gels (M00656; GenScript, USA) alongside a pre-stained protein marker (26616; Thermo Fisher Scientific, USA) and then transferred onto polyvinylidene difluoride (PVDF) membranes (IPVH00010; Merck Millipore, USA) at a constant current of 200 mA for 90 min in an ice bath. Membranes were blocked with 5% skim milk powder (232100; Becton Dickinson, USA) or 5% bovine serum albumin (BSA; S12012; Shyuanye, China) in tris-buffered saline (TBS; T1080; Solarbio, China) for 2 h at room temperature, then incubated overnight at 4 °C with specific primary antibodies listed in Supplementary Table S3. After being washed three times with TBS combined with 0.2% Tween-20 (TBST; P1033; Solarbio, China), the membranes were incubated with appropriate secondary antibodies at room temperature for one hour (Supplementary Table S3). A chemiluminescent substrate (34095; Thermo Fisher Scientific, USA) was added to the membranes, and images were acquired using the ChemiDoc XRS imaging system (Bio-Rad, CA, USA). The gray values of the target bands were quantified with Image J software (NIH, ML, USA) and normalized to GAPDH.

### Transcriptomic analysis

RNA extraction followed the manufacturer’s guidelines, utilizing the RNA Nano 6000 Assay Kit on the Bioanalyzer 2100 system (Agilent Technologies, Santa Clara, USA). RNA integrity and purity were assessed using the 2,100 bioanalyzer and photometer (Agilent Technologies, Santa Clara, USA). Library construction was performed using the NEB Next UltraRNA Library Prep Kit for Illumina (NEB, USA) (Parkhomchuk et al., 2009). Initial quantification was conducted with a Qubit 2.0 fluorometer, and the library, diluted to 1.5 ng/μL, underwent insert size assessment using an Agilent 2100 bioanalyzer. RT-qPCR ensured the effective library concentration exceeded 2 nM. Subsequently, sequencing occurred on the HISEQ4000, 150 PE platform by Novogene Bioinformatics Technology Co., Ltd.

Clean reads, obtained post-filtering from original sequencing data, were used for Q20, Q30, and GC content calculations. HISAT2 v2.0.5 aligned clean reads to the reference genome after quality control analysis. DESeq R package (1.18.0) was employed for differential expression analysis across three groups, each with three biological replicates. Significant DEGs had *P*-value <0.05 and |log2FoldChange| ≥ 1.0. Pathway analysis was conducted using the Kyoto Encyclopedia of Genes and Genomes (KEGG) database, with DEGs considered significantly enriched when the *P*-value <0.05. All visualizations were generated using https://magic.Novogene.com/public/customer and http://www.bioinformatics.com.cn, which are online platforms dedicated to data analysis and visualization. The STRING database was utilized to construct predicted protein–protein interaction (PPI) networks for DEGs, with interactions having combined scores >0.4 considered significant. The resulting networks were then imported into Cytoscape software (version 3.9.1), and hub genes were subsequently identified using the cytoHubba plugin.

### Statistical analysis

The data were presented as means ± standard deviation. Statistical analysis was carried out using SPSS 20.0 (SPSS Inc., Chicago, IL, USA). To determine the statistical significance of measurement data across multiple groups, a one-way analysis of variance (ANOVA) was performed, followed by multiple comparisons with the Duncan’s multiple range test. Statistical significance was defined as *P* < 0.05, while 0.05 ≤ *P* < 0.10 were considered indicative of a trend. Prism software (version 8. 3. 0; GraphPad, San Diego, CA, USA) was used for graph plotting.

## Results

### 18β-Glycyrrhetinic acid alleviates renal D-galactose-induced oxidative stress injury

**Figure 1.**
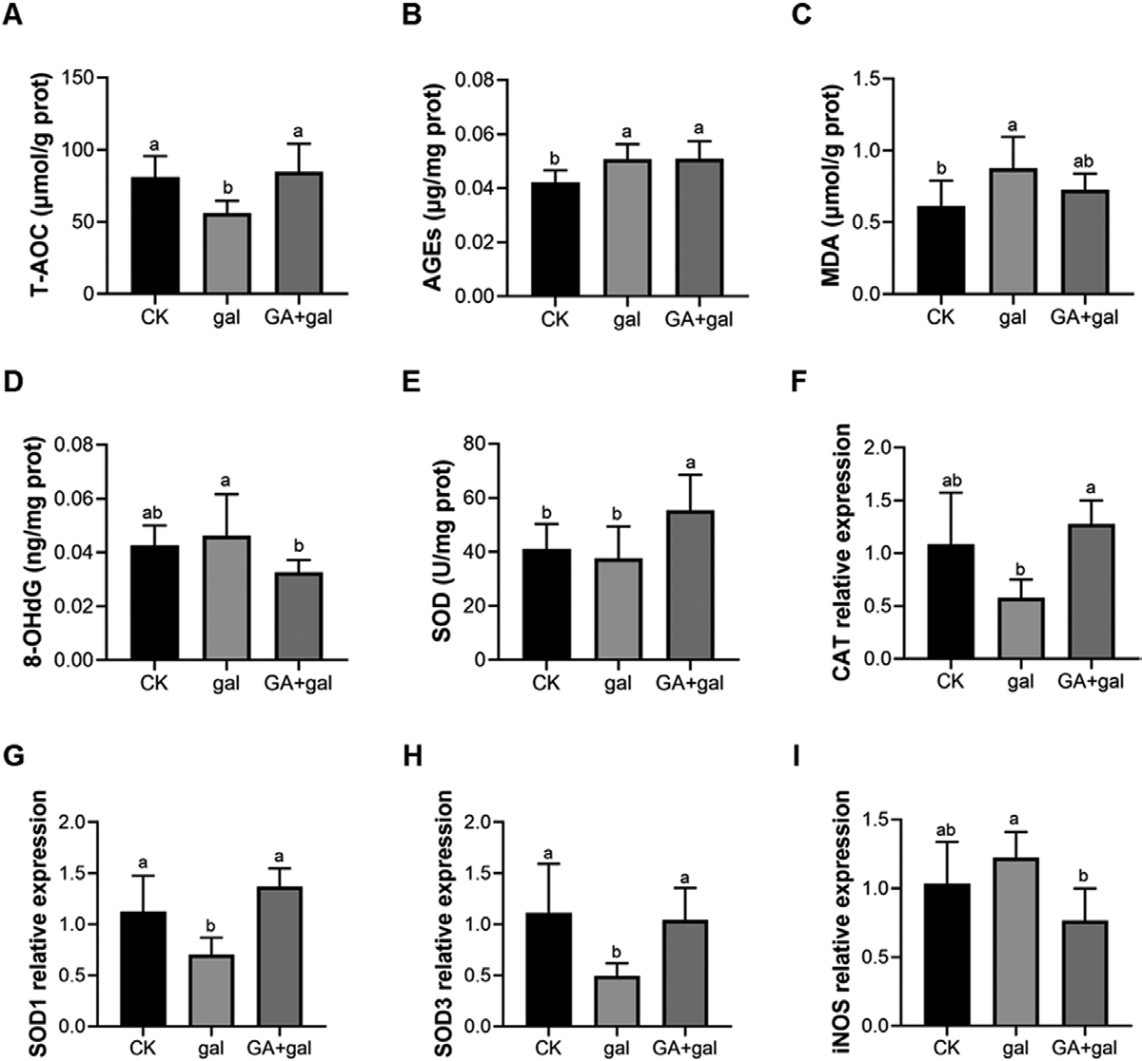
Effect of 18β-glycyrrhetinic acid on antioxidant capacity in D-galactose-treated piglet kidneys. (A) The levels of total antioxidant capacity (T-AOC). The levels of (B) advanced glycation end products (AGEs), (C) malondialdehyde (MDA), and (D) 8-hydroxy-2’-deoxyguanosine (8-OHdG). (E) Activity of SOD (n = 6). Gene expression of (F) *CAT*, (G) *SOD1*, (H) *SOD3*, and (I) *iNOS* (n = 4). Different lowercase letters indicate significant differences between the experimental groups (*P* < 0.05).

D-gal ingestion has been reported to induce systemic oxidative stress in pigs (Li et al., 2020; Han et al., 2021). To assess whether it induces renal oxidative stress, we analyzed oxidative stress biomarkers in the kidneys of piglets. The potential protective effect of GA was also assessed. Compared with the CK group, the gal group exhibited a significant reduction in T-AOC (*P* < 0.05, Figure 1A). GA supplementation significantly restored T-AOC levels (*P* < 0.05, Figure 1A). Furthermore, the gal group demonstrated significantly elevated levels of AGEs and MDA compared with the CK group (*P* < 0.05, Figure 1B and C), both key markers of oxidative damage. GA supplementation normalized MDA levels (Figure 1C). And the levels of 8-OHdG, a marker of DNA oxidative damage, were significantly reduced in the GA + gal group compared with the gal group (*P* < 0.05, Figure 1D). Moreover, GA markedly enhanced SOD activity in the kidneys of D-gal-induced piglets (*P* < 0.05, Figure 1E). It also up-regulated the gene expression of *CAT*, *SOD1*, and *SOD3* (*P* < 0.05, Figure 1F–H) while down-regulating *iNOS* (*P* < 0.05, Figure 1I) in the kidneys of these piglets. These findings demonstrate that D-gal induces oxidative stress in piglet kidneys, while GA effectively mitigates this stress.

### 18β-Glycyrrhetinic acid reduces D-galactose-induced inflammatory response in piglet Kidneys

**Figure 2.**
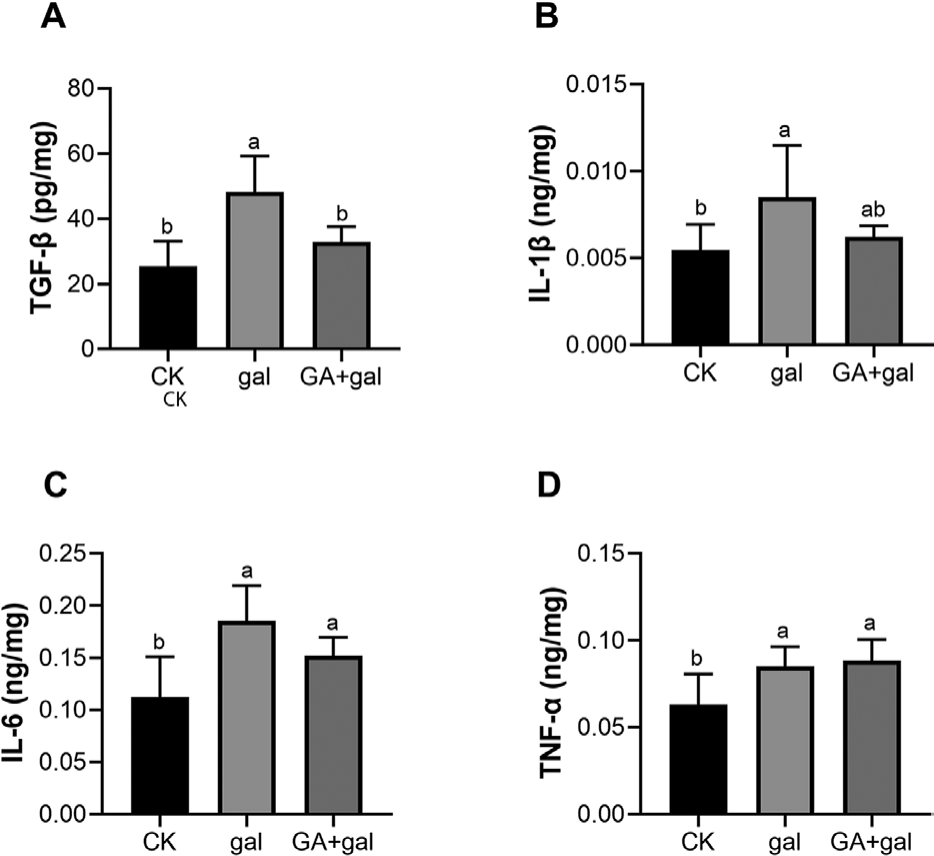
Effect of 18β-glycyrrhetinic acid on inflammatory cytokines in D-galactose-treated piglet kidneys. Levels of inflammatory factors (A) transforming growth factor-β (TGF-β), (B) interleukin 1 beta (IL-1β), (C) interleukin 6 (IL-6), (D) and tumor necrosis factor-alpha (TNF-α). (*n* = 6). Different lowercase letters indicate significant differences between the experimental groups (*P* < 0.05).

Oxidative stress is known to increase cytokine production, with inflammation being a hallmark of oxidative stress-related renal injury (Elmarakby and Sullivan, 2012; Yang et al., 2022). As shown in Figure 2A–D, the levels of TGF-β, IL-1β, IL-6, and TNF-α were significantly higher in the kidneys of the gal group compared to the CK group (*P* < 0.05). Supplementation with GA normalized the levels of TGF-β and IL-1β (Figure 2A and B). These results suggest that GA may play a role in attenuating renal inflammation with D-gal-induced kidney injury.

### Protective role of 18β-glycyrrhetinic acid against D-galactose-induced renal injury

**Figure 3.**
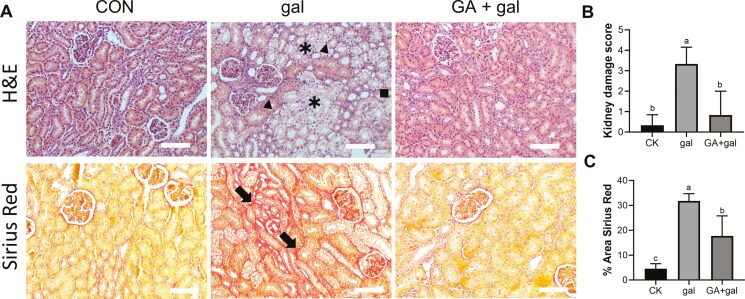
Effect of 18β-glycyrrhetinic acid on renal damage in D-galactose-treated piglet. (A) Representative H&E and Sirius Red stained renal tissue sections from each group (× 200 magnification; scale bars: 100 μm), with asterisks indicating tubular epithelial vacuoles, triangle indicating inflammatory cell infiltration, square indicating loss of brush border, and arrow indicating obvious fibrosis. (B) Scores for kidney injury (0 = none, 1 = 0% to 20%, 2 = 20% to 50%, 3 = 50% to 70%, 4 = more than 70%). (C) Quantification of fibrotic areas. Different lowercase letters indicate significant differences between the experimental groups (*P* < 0.05).

To investigate the potential protective effects of GA against D-gal-induced renal injury, the kidney index was calculated, as depicted in Supplementary Figure S1. The gal group showed a significant decrease in kidney index compared to the CK group (*P* < 0.05), while no difference was observed between the GA + gal and CK groups (*P* > 0.05). Histopathological analysis of piglet kidney sections was conducted, as shown in Figure 3A. The CK group exhibited structurally intact renal tissue with normal tubular morphology. In contrast, D-gal-induced injury caused tubular epithelial vacuolation (indicated by asterisks), increased inflammatory cell infiltration (indicated by triangles), and loss of the brush border (indicated by squares). GA treatment alleviated these pathological changes, restoring renal architecture. Kidney injury scoring demonstrated that GA supplementation significantly reduced the damage score compared to the gal group (*P* < 0.05, Figure 3B). Sirius Red staining further revealed that fibrotic deposition in the kidneys was significantly decreased in the GA + gal group compared to the gal group (*P* < 0.05, Figure 3C). These findings suggest that GA offers a protective effect against D-gal-induced renal injury and improves renal fibrosis.

### Transcriptome profile analysis of the kidney tissue

#### DEGs involved in antioxidant, and anti-Inflammatory responses.

To explore the gene expression profiles associated with the pathological changes observed in piglet kidneys, a transcriptome profiling analysis was conducted. The total number of reads obtained is presented in Supplementary Table S4. In the GA + gal group, antioxidant genes such as *SOD3* and *GSTA1* were upregulated. Meanwhile, inflammation-related gene *IL1R1* and fibrosis-related genes, including *FBLN2*, *TGFB1I1*, *GDF7*, and *COL1A1*, were downregulated. These changes support GA’s protective effects in enhancing antioxidant, anti-inflammatory, and antifibrotic responses.

#### PPI network highlights key genes associated with renal fibrosis.

**Figure 4.**
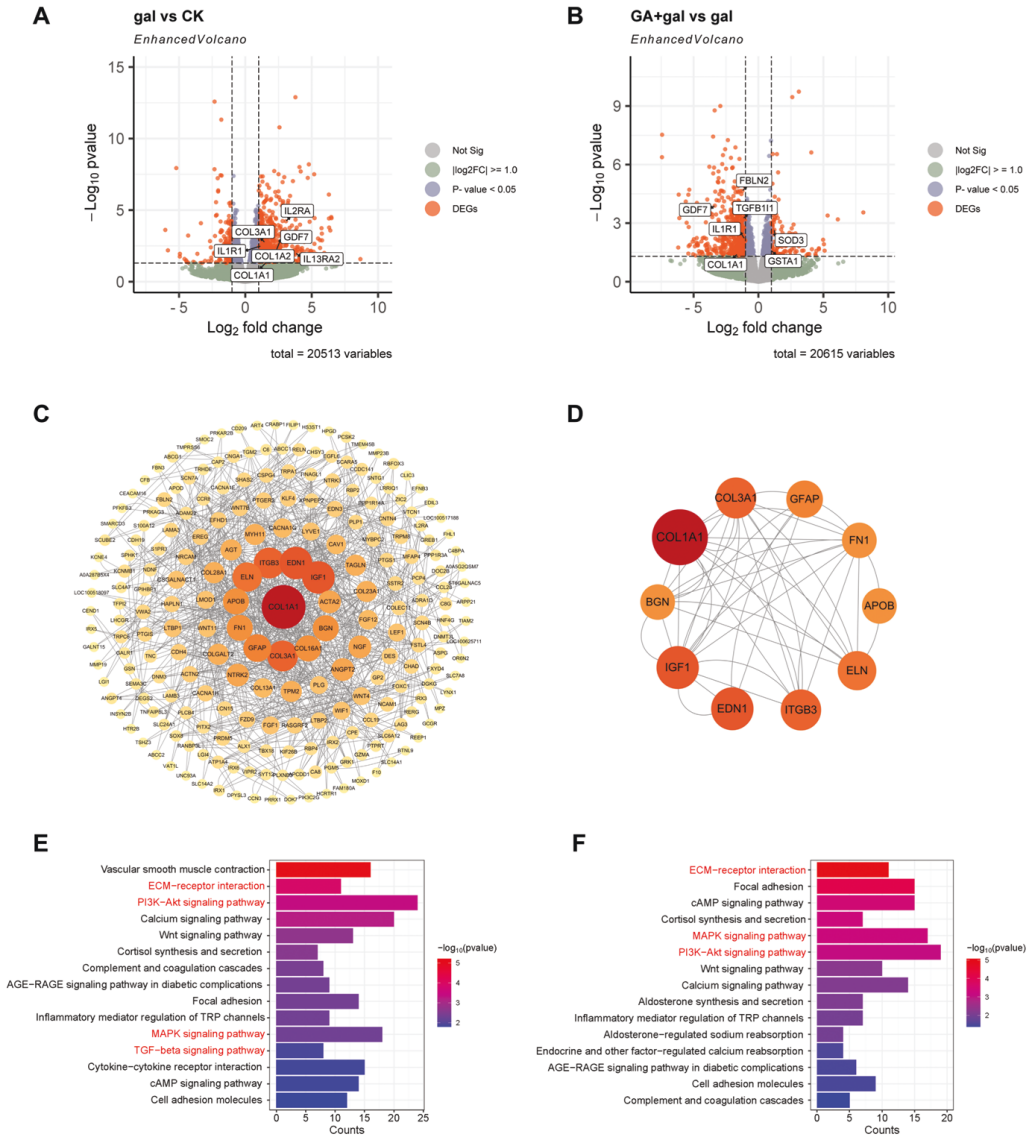
Functional analysis of differentially expressed genes (DEGs) following treatment with D-galactose and 18β-glycyrrhetinic acid. The Volcano plots highlight DEGs in (A) gal vs. CK and (B) GA + gal vs. gal. (C) Protein-protein interaction (PPI) network of shared DEGs identified in the gal vs. CK and GA + gal vs. gal comparisons, with nodes representing genes and edges representing interactions. Node size and the number of connecting edges indicate the degree of interaction. (D) Identification of 10 hub genes within the PPI network. (E) KEGG enrichment analyses of (E) up-regulated DEGs in gal vs. CK and (F) down-regulated DEGs in GA + gal vs. gal, respectively.

A PPI network was constructed based on 409 common DEGs from the CK vs. gal and GA + gal vs. gal comparisons to identify functionally relevant proteins (Supplementary Figure S2). The network nodes were arranged by their degree of interaction, as shown in Figure 4C. The top 10 hub genes identified within this network are displayed in Figure 4D: *COL1A1*, *EDN1*, *IGF1*, *COL3A1*, *ITGB3*, *ELN*, *GFAP*, *APOB*, *BGN*, and *FN1*. Functional annotation of these genes revealed that most are associated with renal fibrosis (Li et al., 2021; Dou et al., 2023). These findings suggest that the 10 hub genes are critical targets of GA treatment for alleviating D-gal-induced renal fibrosis and may serve as potential therapeutic targets.

#### KEGG pathway enrichment analysis of DEGs.

KEGG enrichment analysis revealed pathways related to oxidative stress, inflammation, and fibrosis progression that were enriched in upregulated DEGs from the gal vs. CK comparison and downregulated DEGs from the GA + gal vs. gal comparison (Figure 4E and F). All significantly enriched pathways are provided in Supplementary Tables S5 and S6. These results suggest that key pathways, including ECM–receptor interaction, PI3K-AKT signaling, MAPK signaling, and TGF-beta signaling, may play a crucial role in the antioxidant and anti-inflammatory effects observed following GA treatment. To validate the RNA-Seq data, 10 DEGs were randomly selected for RT-qPCR, and the results showed excellent linearity with RNA-Seq, confirming the data’s reliability (Supplementary Figure S3).

### 18β-Glycyrrhetinic acid alleviates D-galactose-induced renal injury via TGF-β/PI3K/AKT, p38 MAPK and Nrf2 signaling pathways

**Figure 5.**
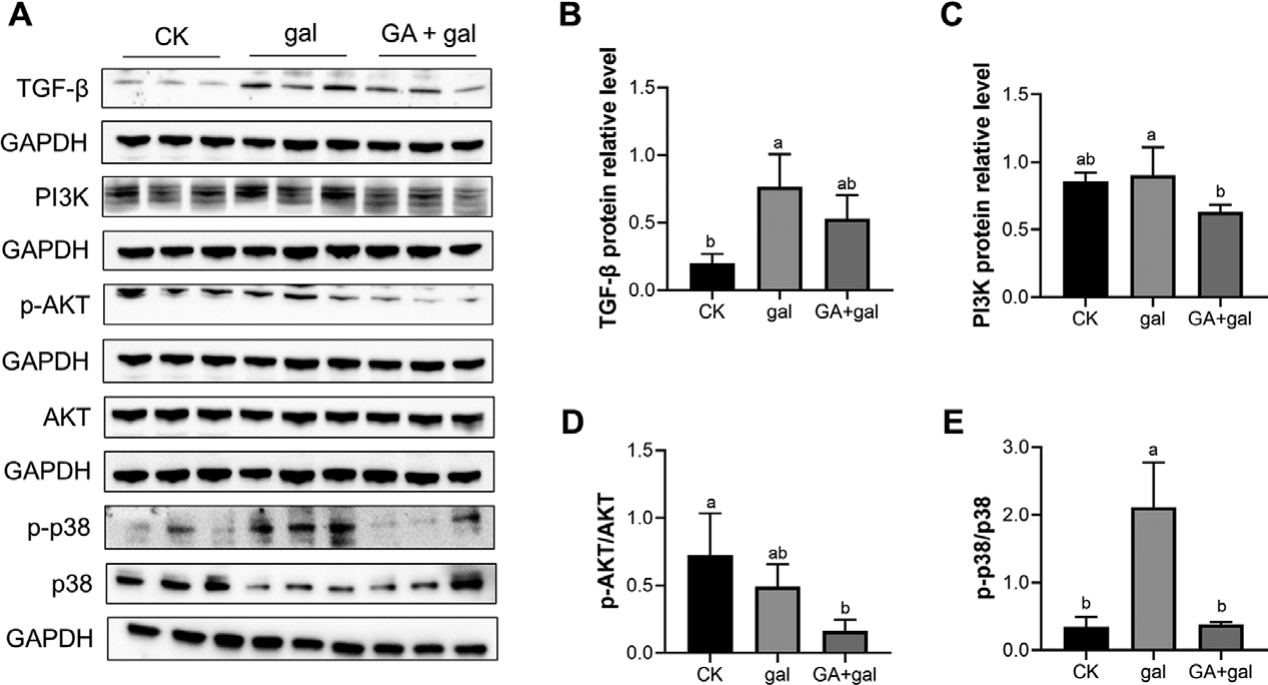
Effect of 18β-glycyrrhetinic acid on the TGF-β/PI3K/AKT and p38 MAPK signaling pathways in D-galactose-treated piglet kidneys. (A) Protein immunoblotting. Protein expression levels of (B) TGF-β, (C) PI3K, (D) p-AKT/AKT, and (E) p-p38/p38, normalized to GAPDH (*n* = 3). Different lowercase letters indicate significant differences between the experimental groups (*P *< 0.05).

GA treatment restored TGF-β1 protein levels to normal (Figure 5B). Compared with the gal group, PI3K protein levels were significantly reduced in the GA + gal group (*P* < 0.05, Figure 5C), and p-AKT/AKT protein levels exhibited a decreasing trend (*P* = 0.099, Figure 5D). Additionally, phosphorylation of p38 MAPK was markedly elevated in the gal group relative to controls (*P* < 0.05), while GA supplementation significantly attenuated p38 phosphorylation (*P* < 0.05, Figure 5E). These results corroborate the transcriptomic findings, indicating that downregulation of the TGF-β/PI3K/AKT and p38 MAPK pathways contributes to the protective effects of GA against D-galactose-induced inflammation and renal fibrosis.

**Figure 6.**
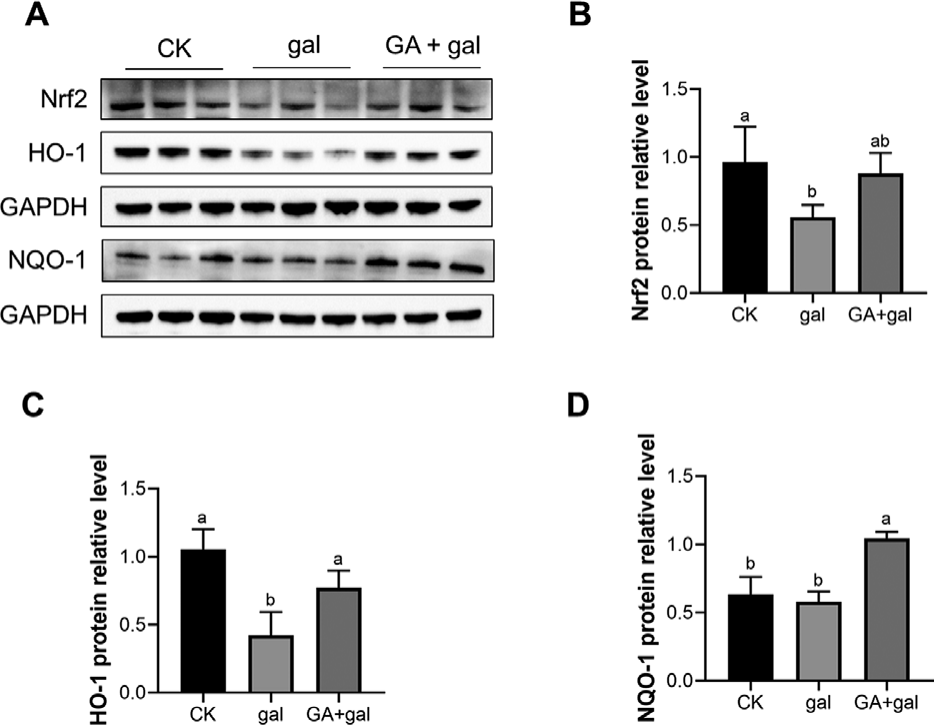
Effect of 18β-glycyrrhetinic acid on Nrf2 signaling pathway in D-galactose-treated piglet kidneys. (A) Protein immunoblotting. Protein expression levels of (B) Nrf2, (C) HO-1, and (D) NQO-1, normalized to GAPDH (*n* = 3). Different lowercase letters indicate significant differences between the experimental groups (*P *< 0.05).

Extensive research underscores the central role of the Nrf2 signaling pathway in mitigating oxidative stress (Wang et al., 2019). Although this pathway was not directly identified in the transcriptomic analysis, the up-regulation of downstream antioxidant enzymes *SOD3* and *GSTA1* suggests potential activation, warranting further investigation into its role in GA treatment. Protein expression of Nrf2 showed an increasing trend in the GA + gal group compared with the gal group (*P* = 0.071, Figure 6B), while levels of HO-1 and NQO-1 were significantly elevated (*P* < 0.05, Figure 6C and D). These results suggest that GA activates the Nrf2 signaling pathway, thereby enhancing antioxidant defenses and ultimately alleviating D-galactose-induced renal injury in piglets.

## Discussion

As a primary regulator of systemic homeostasis, renal health is crucial for maintaining physiological function and supporting optimal growth performance in swine. However, common oxidative challenges in pig production, such as weaning, can induce renal oxidative damage. This damage impedes detoxification processes, leading to multisystemic dysregulation including electrolyte imbalance, uremic toxin accumulation, and endocrine dysfunction (Piko et al., 2023). These disturbances may reduce feed efficiency, weaken immune function, and ultimately compromise overall production performance (Marin et al., 2017; Dos Santos Cruz et al., 2022). In this study, D-gal treatment successfully induced renal oxidative damage, as indicated by increased AGEs and MDA levels and characteristic histopathological lesions. Supplementation with GA effectively mitigated this damage by enhancing antioxidant capacity, as evidenced by increased T-AOC and SOD levels and reduced 8-OHdG concentrations. GA also reversed the D-gal-induced elevation of TGF-β. Mechanistically, GA downregulated the TGF-β/PI3K/AKT pathway to reduce ECM accumulation and fibrosis, suppressed p38 MAPK signaling to alleviate inflammation, and activated the Nrf2 pathway to enhance renal antioxidant defenses. These findings suggest that dietary GA supplementation helps maintain renal health in weaned piglets under oxidative stress, thereby improving their resilience to weaning challenges and supporting production efficiency.

Renal injury induced by D-gal is primarily caused by oxidative damage, a mechanism extensively documented (Sifuentes-Franco et al., 2018; Chen et al., 2020). In this study, D-gal administration induced oxidative renal injury, as evidenced histologically by tubular epithelial vacuolization, brush border disintegration, inflammatory infiltration, and fibrosis (Abdel-Daim et al., 2021; Zhang et al., 2021). GA supplementation enhanced renal antioxidant capacity, as shown by increased T-AOC levels, elevated SOD activity, and upregulation of *CAT*, *SOD1*, and *SOD3* genes, aligning with previous studies indicating that GA restores the balance between pro-oxidants and antioxidants (Cheng et al., 2020; Zhou et al., 2021). In addition to oxidative stress, inflammation plays a crucial role in D-gal-induced renal injury, as the relationship between oxidative stress and inflammation is well established (Lugrin et al., 2014). Elevated levels of inflammatory markers, including TGF-β, IL-1β, IL-6, and TNF-α, were observed in D-gal-treated kidneys, accompanied by infiltration of inflammatory cells, as revealed by H&E staining. GA efficiently modulated inflammatory responses by restoring TGF-β and IL-1β levels. Furthermore, Sirius Red staining confirmed that GA significantly attenuated renal fibrosis, a late consequence of chronic inflammation and oxidative stress (Liu, 2011). These results suggest that GA not only mitigates initial oxidative damage and inflammation but also protects against the development of renal fibrosis.

TGF-β1, a well-characterized cytokine, plays a critical role in renal inflammation and fibrosis by promoting ECM production while inhibiting ECM degradation (Meng et al., 2016; Larson et al., 2020). In addition, TGF-β1 induces epithnelial–mesenchymal transition, which converts tubular epithelial cells into myofibroblasts, a critical step in the progression of fibrosis (Lan, 2011). Our results showed that GA inhibited *TGFB1I1* expression and downregulated ECM-receptor interactions in porcine kidney tissue. Transcriptomic analysis further revealed that GA suppressed the PI3K/AKT signaling pathway in D-gal-induced kidneys. Previous studies indicated that TGF-β activation can drive renal interstitial fibrosis through both classical and non-classical pathways. In the classical pathway, TGF-β/Smad signaling activates myofibroblasts and promotes ECM deposition via a Smad-2/3/4 complex (Nakao et al., 1997; Rosenbloom et al., 2010). Conversely, the non-classical pathway involves effectors such as PI3K/AKT, c-Abl, PAK2, and mTOR (Wang et al., 2005; Feng et al., 2022). Western blot analysis confirmed that GA alleviated renal fibrosis by targeting the TGF-β/PI3K/AKT pathway. Additionally, transcriptomic analysis showed that GA suppressed MAPK signaling pathway activation induced by D-gal. Consistent with this, Western blot results confirmed that GA significantly reduced p38 MAPK phosphorylation, thereby attenuating the renal inflammatory response (Yan et al., 2025). This anti-inflammatory effect complements its antifibrotic actions and contributes to the overall renoprotective effects of GA.

Previous research has demonstrated that GA targets specific genes involved in oxidative stress regulation, including *Nrf2*, *HO-1*, and *PPARs* (Mahmoud and Al Dera, 2015; Cheng et al., 2022). Nrf2, a basic leucine zipper protein encoded by the *NFE2L2* gene, regulates the expression of antioxidant genes (Wang et al., 2023). Under normal conditions, Nrf2 is sequestered in the cytoplasm and targeted for Keap1-mediated ubiquitination and proteasomal degradation. However, during oxidative stress, Nrf2 evades degradation, stabilizes, and translocates to the nucleus, where it activates the transcription of antioxidant genes such as *SOD*, *GST*, *HO-1*, and *NQO1* through the antioxidant response element (Park et al., 2015; Bellezza et al., 2018). This regulatory axis highlights the critical role of Nrf2 in cellular defense against oxidative stress (Itoh et al., 2010). Consistent with these findings, Wu et al. demonstrated that GA reduced cisplatin-induced renal oxidative stress by upregulating Nrf2 expression (Wu et al., 2015). In the present study, we observed that GA significantly upregulated mRNA levels of Nrf2 downstream targets (*SOD3*, *GSTA1*), despite the absence of significant changes in Nrf2 signaling pathway expression in RNA-seq data. Western blot analysis further revealed that GA supplementation significantly increased the protein levels of downstream antioxidant enzymes, including HO-1 and NQO1, and showed an increasing trend in Nrf2 expression. This apparent discrepancy may reflect post-transcriptional regulatory mechanisms, as Nrf2 activation is primarily governed by protein-level modifications rather than transcriptional upregulation (Bloom and Jaiswal, 2003). Collectively, these findings suggest that GA attenuates renal oxidative damage through a multifaceted mechanism involving activation of the Nrf2 signaling pathway and inhibition of the TGF-β/PI3K/AKT and p38 MAPK signaling pathways.

## Conclusion

In conclusion, our study demonstrates that GA effectively alleviates symptoms of D-gal-induced renal oxidative stress and inflammation in piglets, while also inhibiting the progression of renal fibrosis. Specifically, GA supplementation significantly increased T-AOC levels, reduced 8-OHdG levels, increased SOD activity, and upregulated antioxidant genes (*CAT*, *SOD1*, *SOD3*), while downregulating *iNOS*. Furthermore, GA reversed the elevated levels of TGF-β and IL-1β induced by D-gal, restored renal structure, and alleviated fibrosis. Mechanistically, GA downregulated the TGF-β/PI3K/AKT pathway to attenuate ECM accumulation and renal fibrosis, and suppressed the p38 MAPK pathway to mitigate inflammation. Additionally, GA enhanced renal antioxidant capacity by activating the Nrf2 signaling pathway, thereby protecting against oxidative damage. As a natural plant extract used as a feed supplement, GA holds substantial potential for enhancing renal health and may serve as a promising strategy for preventing oxidative damage in livestock.

## Supplementary Material

skaf240_suppl_Supplementary_Materials_1

## Abbreviations

8-OHdG: 8-hydroxy-2’ -deoxyguanosine
ADFI: average daily feed intake
ADG: average daily gain
AGEs: Advanced glycation end products
AKT: protein kinase B
ANOVA: Analysis of Variance
ARE: antioxidant response element
BCA: bicinchoninic acid
BW: body weight
D-gal: D-galactose
ECM: excessive extracellular matrix
EMT: epithnelial-mesenchymal transition
F/G: feed to gain ratio
GA: 18β-glycyrrhetinic acid
GADPH: glyceraldehyde-3-phosphate dehydrogenase
H&E: hematoxylin and eosin
HO-1: heme oxygenase-1
IL-1β: interleukin 1beta
IL-6: interleukin 6
Keap1: Kelch ECH-associating protein 1
KEGG: Kyoto Encyclopedia of Genes and Genomes
MDA: malondialdehyde
NF-κB: nuclear factor kappa-light-chain-enhancer of activated B cells
NQO-1: NAD(P)H quinone dehydrogenase 1
Nrf2: nuclear factor erythroid 2-related factor 2
p-AKT: phosphorylated protein kinase B
PFA: paraformaldehyde
PI3K: phosphoinositide 3-kinase
PPI: protein-protein interaction
PVDF: polyvinylidene difluoride
RNS: reactive nitrogen species
ROS: reactive oxygen species
RT-qPCR: quantitative real-time PCR
SDS-PAGE: SDS-polyacrylamide gel electrophoresis
SMAD3: mothers against decapentaplegic homolog 3
SOD: superoxide dismutase
T-AOC: total antioxidant capacity
TBST: Tris-buffered saline combined with 0.2% Tween-20
TGF-β: transforming growth factor-β
TNF-α: tumor necrosis factor-alpha
