# Supplementary material for: Supplementation of 18β-glycyrrhetinic acid attenuates D-galactose-induced oxidative stress and inflammatory responses in kidneys of weaned piglet
Source: J Anim Sci. 2025 Jul 31;103:skaf240. doi: 10.1093/jas/skaf240 (PMC12448410; doi:10.1093/jas/skaf240)
Supplement: skaf240_suppl_Supplementary_Materials_1 [file skaf240_suppl_supplementary_materials_1.docx]

Table S1 Composition and nutrient levels of the basal diets (dry matter, %)^1^

| Item | Contents, % |
| --- | --- |
| Ingredient |  |
| Corn | 42.83 |
| Extruded corn | 12.00 |
| 46% Soybean meal | 11.00 |
| Extruded soybean | 8.80 |
| Soybean protein concentrate | 4.50 |
| Driedwhey | 10.00 |
| Super fish meal | 3.10 |
| Soybean oil | 0.60 |
| Limestone | 0.65 |
| Dicalcium phosphate | 1.20 |
| NaCl | 0.30 |
| Glucose | 3.00 |
| 98.5 Ly*s* | 0.54 |
| 98.5% Met | 0.22 |
| Thr | 0.21 |
| Trp | 0.05 |
| Premix^2^ | 1.00 |
| Calculated composition^3^ |  |
| NE, MJ/kg | 10.58 |
| CP | 18.92 |
| Ca | 1.21 |
| SID^4^ Lys | 1.41 |
| SID Met | 0.54 |
| SID Thr | 0.85 |
| SID Trp | 0.25 |
| SID Met+Cys | 0.85 |

^1^A single basal diet was fed to all piglets throughout the 28-day experimental period, and the feed was provided in mash form.

^2^Premix was provided the following per kilogram of diet: 12 000 IU of vitamin A, 4.3 mg of vitamin B_1_, 12 mg of vitamin B_2_, 15 mg of vitamin B_5_, 4.86 mg of vitamin B_6_, 0.48 mg of vitamin B_7_, 1.5 mg of vitamin B_9_, 30 ug of vitamin B_12_, 3000 IU of vitamin D_3_, 16 IU of vitamin E, 4.5 mg of vitamin K_3_, 400 mg of choline chloride, 5 mg of Cu as copper sulfate, 140 mg of Fe as ferrous sulfate, 100 mg of Zn as zinc sulfate, 40 mg of Mn as manganese sulfate, 0.15 mg of I as potassium iodide, and 0.30 mg of Se as sodium selenite.

^3^Nutrient levels were calculated values, except for CP and Ca, which were actual measured values.

^4^Standardized ileal digestible. Coefficients were taken from NRC (2012).

Table S2 Primer sequences for qPCR analysis.

| Target gene | Forward (5' to 3') | Reverse (5' to 3') | Product length/bp | GeneBank accession No. |
| --- | --- | --- | --- | --- |
| *GAPDH* | TGTCCACCTTCCAGCAGATGT | AGCTCAGTAACAGTCCGCCTAGA | 132 | NM_001206359.1 |
| *CAT* | ACGCCTGTGTGAGAACATTG | GTCCAGAAGAGCCTGAATGC | 124 | NM_214301.2 |
| *SOD1* | TCCATGTCCATCAGTTTGGA | AGTCACATTGCCCAGGTCTC | 131 | NM_001190422.1 |
| *SOD3* | ACGCTGCTCTGTGCTTACCT | CTGCCAGATCTCCGTCACTT | 135 | NM_001078688.1 |
| *iNOS* | GGGTCAGAGCTACCATCCTC | CGTCCATGCAGAGAACCTTG | 114 | XM_013981166.2 |
| *INSR* | TATTCATCCCCAGGCCATCCAG | TTGGAGAAACCCGGAATGGT | 91 | XM_021083943.1 |
| *GSN* | TCGCCTGCTCCAACAAGATT | GATTAGCTGGGTCCGTCTCG | 204 | XR_002337473.1 |
| *ERBB4* | AGTACCGAGCCTTGCGAAAA | GAGCCACCAACACATAGCCT | 138 | XM_021075968.1 |
| *EMP1* | CTTGGCTCATGCTAACCTCG | TTGGCTTTCCGATGGCTTGT | 211 | XM_021090850.1 |
| *GALNT15* | TCACTAGGAGATGCCCAATGC | GCCTTTGCACTCTCTTCACAC | 220 | XM_021068276.1 |
| *ADGRB1* | ACCTTTCTCACCTAAGCCGC | ATAGATTCCCCACCCCTGCT | 75 | XM_021088791.1 |
| *C6* | CAACAAGCCACAGGTTTCGG | TATGGCTGCTGGTTTGGCTT | 88 | NM_001097449.1 |
| *EGF* | TACTCCACCCTCTCACCTCG | CGCCAACGTAGCCAAAAACA | 164 | NM_214020.2 |
| *TGF-β* | ACGTGGAGCTATACCAGAAATACAG | ACAACTCCGTGACATCAAAGG | 111 | NM_214015.2 |
| *TNF-α* | CACGCTCTTCTGCCTACTGC | GTGGCTCGGCTTTGACATT | 164 | NM_214022.1 |
| *IL-1β* | GTTCTCTGAGAAATGGGAGC | CTGGTCATCATCACAGAAGG | 143 | NM_214055.1 |

Table S3 Antibodies used in this study

| Antibodies | Catalog number | Dilution | RRID | Source |
| --- | --- | --- | --- | --- |
| anti-TGF-β1 | ab92486 | 1:1000 | AB_10562492 | Abcam |
| anti-Nrf2 | ab92946 | 1:1000 | AB_10561604 | Abcam |
| anti-SMAD3 | 9523S | 1:1000 | AB_2193182 | Cell Signaling Technology |
| anti-PI3K | 3811S | 1:1000 | AB_2062856 | Cell Signaling Technology |
| anti-AKT | 9272S | 1:1000 | AB_329827 | Cell Signaling Technology |
| anti-p-AKT | 9271S | 1:1000 | AB_329825 | Cell Signaling Technology |
| anti-phospho-p38 MAPK | 4511T | 1:1000 | AB_2139682 | Cell Signaling Technology |
| anti-GADPH | 2118S | 1:1000 | AB_561053 | Cell Signaling Technology |
| anti-p38 MAPK | 14064-1-AP | 1:1000 | AB_2878007 | Proteintech |
| anti-HO-1 | 66743-1-Ig | 1:1000 | AB_2882091 | Proteintech |
| anti-NQO-1 | 11451-1-AP | 1:1000 | AB_2298729 | Proteintech |
| HRP-Goat Anti-Rabbit IgG (H+L) | HX2031 | 1:5000 | AB_3572247 | Huaxingbio |
| HRP-Goat Anti-Mouse IgG(H+L) | HX2032 | 1:5000 | AB_3662679 | Huaxingbio |

Table S4 Summary of transcriptomic sequencing data quality for samples

| Sample | Raw reads | Raw bases | Clean reads | Clean bases | Q20 (%) | Q30 (%) | GC content (%) | Total mapped | Uniquely mapped |
| --- | --- | --- | --- | --- | --- | --- | --- | --- | --- |
| CK_1 | 44755274 | 6.71 G | 42945798 | 6.44 G | 97.52 | 93.48 | 51.83 | 39880082 (92.86%) | 38904938 (90.59%) |
| CK_2 | 42273366 | 6.34 G | 40868632 | 6.13 G | 97.32 | 93.03 | 51.95 | 37996377 (92.97%) | 37086393 (90.75%) |
| CK_3 | 41790982 | 6.27 G | 40072836 | 6.01 G | 97.49 | 93.46 | 51.55 | 37344912 (93.19%) | 36366766 (90.75%) |
| gal_1 | 41376912 | 6.21 G | 40230680 | 6.03 G | 97.39 | 93.19 | 50.00 | 37389974 (92.94%) | 36530201 (90.8%) |
| gal_2 | 44647248 | 6.70 G | 42936266 | 6.44 G | 97.38 | 93.18 | 51.12 | 39695689 (92.45%) | 38825143 (90.43%) |
| gal_3 | 47284604 | 7.09 G | 44600244 | 6.69 G | 97.41 | 93.29 | 51.33 | 41244535 (92.48%) | 40296367 (90.35%) |
| GA+gal_1 | 42340454 | 6.35 G | 41207386 | 6.18 G | 97.58 | 93.56 | 50.37 | 37752659 (91.62%) | 36891209 (89.53%) |
| GA+gal_2 | 41358960 | 6.20 G | 39917648 | 5.99 G | 97.49 | 93.38 | 51.29 | 37016880 (92.73%) | 36144401 (90.55%) |
| GA+gal_3 | 39563480 | 5.93 G | 38387014 | 5.76 G | 97.54 | 93.53 | 50.46 | 35594938 (92.73%) | 34777774 (90.6%) |

Table S5 KEGG pathway enrichment analysis of up-regulated DEGs in the gal vs. CK comparison.

| Term | Database | ID | p*-*value | Count |
| --- | --- | --- | --- | --- |
| Amoebiasis | KEGG PATHWAY | ssc05146 | 0.0000 | 15 |
| Vascular smooth muscle contraction | KEGG PATHWAY | ssc04270 | 0.0000 | 16 |
| Neuroactive ligand-receptor interaction | KEGG PATHWAY | ssc04080 | 0.0000 | 27 |
| Protein digestion and absorption | KEGG PATHWAY | ssc04974 | 0.0000 | 13 |
| ECM-receptor interaction | KEGG PATHWAY | ssc04512 | 0.0001 | 11 |
| Cushing syndrome | KEGG PATHWAY | ssc04934 | 0.0003 | 14 |
| PI3K-Akt signaling pathway | KEGG PATHWAY | ssc04151 | 0.0004 | 24 |
| Platelet activation | KEGG PATHWAY | ssc04611 | 0.0005 | 12 |
| Basal cell carcinoma | KEGG PATHWAY | ssc05217 | 0.0006 | 8 |
| Calcium signaling pathway | KEGG PATHWAY | ssc04020 | 0.0006 | 20 |
| Proteoglycans in cancer | KEGG PATHWAY | ssc05205 | 0.0009 | 16 |
| Melanogenesis | KEGG PATHWAY | ssc04916 | 0.0011 | 10 |
| Wnt signaling pathway | KEGG PATHWAY | ssc04310 | 0.0020 | 13 |
| Cortisol synthesis and secretion | KEGG PATHWAY | ssc04927 | 0.0026 | 7 |
| Leishmaniasis | KEGG PATHWAY | ssc05140 | 0.0034 | 8 |
| Complement and coagulation cascades | KEGG PATHWAY | ssc04610 | 0.0039 | 8 |
| Human papillomavirus infection | KEGG PATHWAY | ssc05165 | 0.0042 | 20 |
| AGE-RAGE signaling pathway in diabetic complications | KEGG PATHWAY | ssc04933 | 0.0046 | 9 |
| Focal adhesion | KEGG PATHWAY | ssc04510 | 0.0047 | 14 |
| Inflammatory mediator regulation of TRP channels | KEGG PATHWAY | ssc04750 | 0.0049 | 9 |
| MAPK signaling pathway | KEGG PATHWAY | ssc04010 | 0.0050 | 18 |
| Metabolism of xenobiotics by cytochrome P450 | KEGG PATHWAY | ssc00980 | 0.0052 | 6 |
| African trypanosomiasis | KEGG PATHWAY | ssc05143 | 0.0056 | 5 |
| Hematopoietic cell lineage | KEGG PATHWAY | ssc04640 | 0.0056 | 8 |
| Breast cancer | KEGG PATHWAY | ssc05224 | 0.0074 | 11 |
| Serotonergic synapse | KEGG PATHWAY | ssc04726 | 0.0075 | 9 |
| Hypertrophic cardiomyopathy | KEGG PATHWAY | ssc05410 | 0.0078 | 8 |
| Hippo signaling pathway | KEGG PATHWAY | ssc04390 | 0.0097 | 11 |
| Aldosterone synthesis and secretion | KEGG PATHWAY | ssc04925 | 0.0111 | 8 |
| Retinol metabolism | KEGG PATHWAY | ssc00830 | 0.0112 | 6 |
| Viral protein interaction with cytokine and cytokine receptor | KEGG PATHWAY | ssc04061 | 0.0115 | 7 |
| TGF-beta signaling pathway | KEGG PATHWAY | ssc04350 | 0.0131 | 8 |
| Cytokine-cytokine receptor interaction | KEGG PATHWAY | ssc04060 | 0.0146 | 15 |
| cAMP signaling pathway | KEGG PATHWAY | ssc04024 | 0.0150 | 14 |
| Cell adhesion molecules | KEGG PATHWAY | ssc04514 | 0.0161 | 12 |
| Other types of O-glycan biosynthesis | KEGG PATHWAY | ssc00514 | 0.0162 | 5 |
| Chemical carcinogenesis - DNA adducts | KEGG PATHWAY | ssc05204 | 0.0162 | 5 |
| Fluid shear stress and atherosclerosis | KEGG PATHWAY | ssc05418 | 0.0163 | 10 |
| cGMP-PKG signaling pathway | KEGG PATHWAY | ssc04022 | 0.0166 | 11 |
| Drug metabolism - cytochrome P450 | KEGG PATHWAY | ssc00982 | 0.0190 | 5 |
| Phototransduction | KEGG PATHWAY | ssc04744 | 0.0190 | 4 |
| Renin secretion | KEGG PATHWAY | ssc04924 | 0.0207 | 6 |
| Amphetamine addiction | KEGG PATHWAY | ssc05031 | 0.0207 | 6 |
| Circadian entrainment | KEGG PATHWAY | ssc04713 | 0.0208 | 8 |
| Phospholipase D signaling pathway | KEGG PATHWAY | ssc04072 | 0.0243 | 10 |
| Adrenergic signaling in cardiomyocytes | KEGG PATHWAY | ssc04261 | 0.0272 | 10 |
| Dilated cardiomyopathy | KEGG PATHWAY | ssc05414 | 0.0327 | 7 |
| Arrhythmogenic right ventricular cardiomyopathy | KEGG PATHWAY | ssc05412 | 0.0367 | 6 |
| Glycosaminoglycan biosynthesis - chondroitin sulfate / dermatan sulfate | KEGG PATHWAY | ssc00532 | 0.0392 | 3 |
| Signaling pathways regulating pluripotency of stem cells | KEGG PATHWAY | ssc04550 | 0.0411 | 9 |
| Ascorbate and aldarate metabolism | KEGG PATHWAY | ssc00053 | 0.0434 | 3 |
| Pentose and glucuronate interconversions | KEGG PATHWAY | ssc00040 | 0.0477 | 3 |

Table S6 KEGG pathway enrichment analysis of down-regulated DEGs in the GA + gal vs. gal comparison.

| Term | Database | ID | p*-*value | Count |
| --- | --- | --- | --- | --- |
| Protein digestion and absorption | KEGG PATHWAY | ssc04974 | 0.001031 | 12 |
| ECM-receptor interaction | KEGG PATHWAY | ssc04512 | 0.001031 | 11 |
| Neuroactive ligand-receptor interaction | KEGG PATHWAY | ssc04080 | 0.002396 | 22 |
| Cushing syndrome | KEGG PATHWAY | ssc04934 | 0.003007 | 13 |
| Focal adhesion | KEGG PATHWAY | ssc04510 | 0.003895 | 15 |
| Human papillomavirus infection | KEGG PATHWAY | ssc05165 | 0.004273 | 20 |
| Hypertrophic cardiomyopathy | KEGG PATHWAY | ssc05410 | 0.009423 | 9 |
| cAMP signaling pathway | KEGG PATHWAY | ssc04024 | 0.011155 | 15 |
| Cortisol synthesis and secretion | KEGG PATHWAY | ssc04927 | 0.011733 | 7 |
| MAPK signaling pathway | KEGG PATHWAY | ssc04010 | 0.011733 | 17 |
| Basal cell carcinoma | KEGG PATHWAY | ssc05217 | 0.011733 | 7 |
| PI3K-Akt signaling pathway | KEGG PATHWAY | ssc04151 | 0.016141 | 19 |
| Adrenergic signaling in cardiomyocytes | KEGG PATHWAY | ssc04261 | 0.022434 | 11 |
| Melanogenesis | KEGG PATHWAY | ssc04916 | 0.037525 | 8 |
| Arrhythmogenic right ventricular cardiomyopathy | KEGG PATHWAY | ssc05412 | 0.037525 | 7 |
| Amoebiasis | KEGG PATHWAY | ssc05146 | 0.037525 | 8 |
| Hippo signaling pathway | KEGG PATHWAY | ssc04390 | 0.04979 | 10 |
| Vascular smooth muscle contraction | KEGG PATHWAY | ssc04270 | 0.067578 | 9 |
| Wnt signaling pathway | KEGG PATHWAY | ssc04310 | 0.068361 | 10 |
| Calcium signaling pathway | KEGG PATHWAY | ssc04020 | 0.083916 | 14 |
| Aldosterone synthesis and secretion | KEGG PATHWAY | ssc04925 | 0.083916 | 7 |
| Dilated cardiomyopathy | KEGG PATHWAY | ssc05414 | 0.083916 | 7 |
| Breast cancer | KEGG PATHWAY | ssc05224 | 0.088829 | 9 |
| Proteoglycans in cancer | KEGG PATHWAY | ssc05205 | 0.093904 | 11 |
| Synaptic vesicle cycle | KEGG PATHWAY | ssc04721 | 0.093904 | 6 |
| Inflammatory mediator regulation of TRP channels | KEGG PATHWAY | ssc04750 | 0.093904 | 7 |
| Aldosterone-regulated sodium reabsorption | KEGG PATHWAY | ssc04960 | 0.093904 | 4 |
| Signaling pathways regulating pluripotency of stem cells | KEGG PATHWAY | ssc04550 | 0.188037 | 8 |
| Ovarian steroidogenesis | KEGG PATHWAY | ssc04913 | 0.193493 | 4 |
| Axon guidance | KEGG PATHWAY | ssc04360 | 0.209813 | 9 |
| Endocrine and other factor-regulated calcium reabsorption | KEGG PATHWAY | ssc04961 | 0.234928 | 4 |
| AGE-RAGE signaling pathway in diabetic complications | KEGG PATHWAY | ssc04933 | 0.244083 | 6 |
| Cell adhesion molecules | KEGG PATHWAY | ssc04514 | 0.247955 | 9 |
| Gastric acid secretion | KEGG PATHWAY | ssc04971 | 0.274886 | 5 |
| Phototransduction | KEGG PATHWAY | ssc04744 | 0.283425 | 3 |
| Complement and coagulation cascades | KEGG PATHWAY | ssc04610 | 0.283425 | 5 |
| Serotonergic synapse | KEGG PATHWAY | ssc04726 | 0.286235 | 6 |


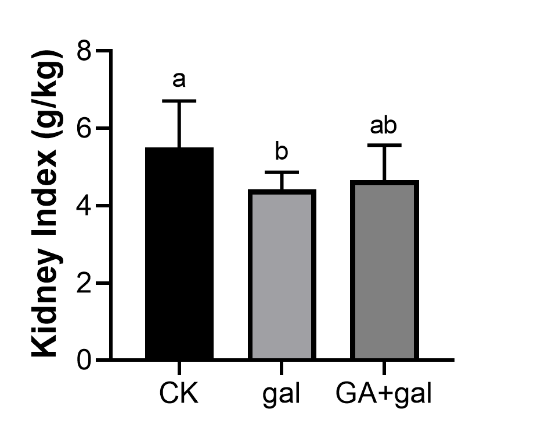


Figure S1 Kidney index determined as the ratio of kidney weight (g) to body weight (kg). (n = 8) Different lowercase letters indicate significant differences between the experimental groups (*P <*0.05).


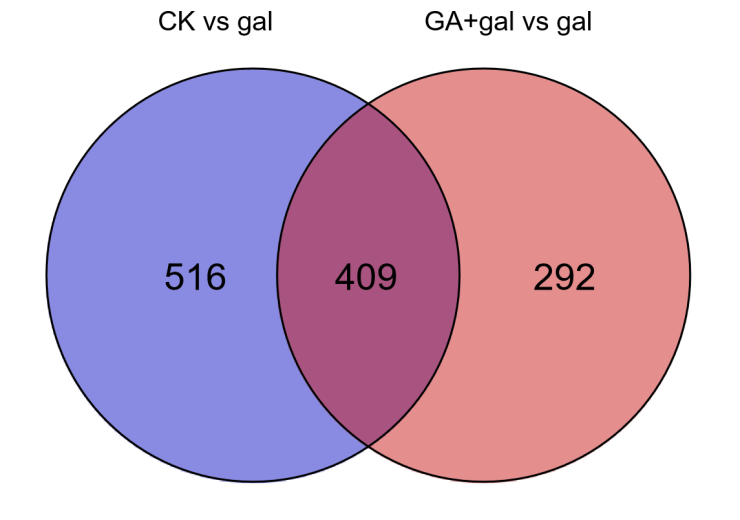


Figure S2 The quantity and intersection of differentially-expressed genes (DEGs) among the three groups.


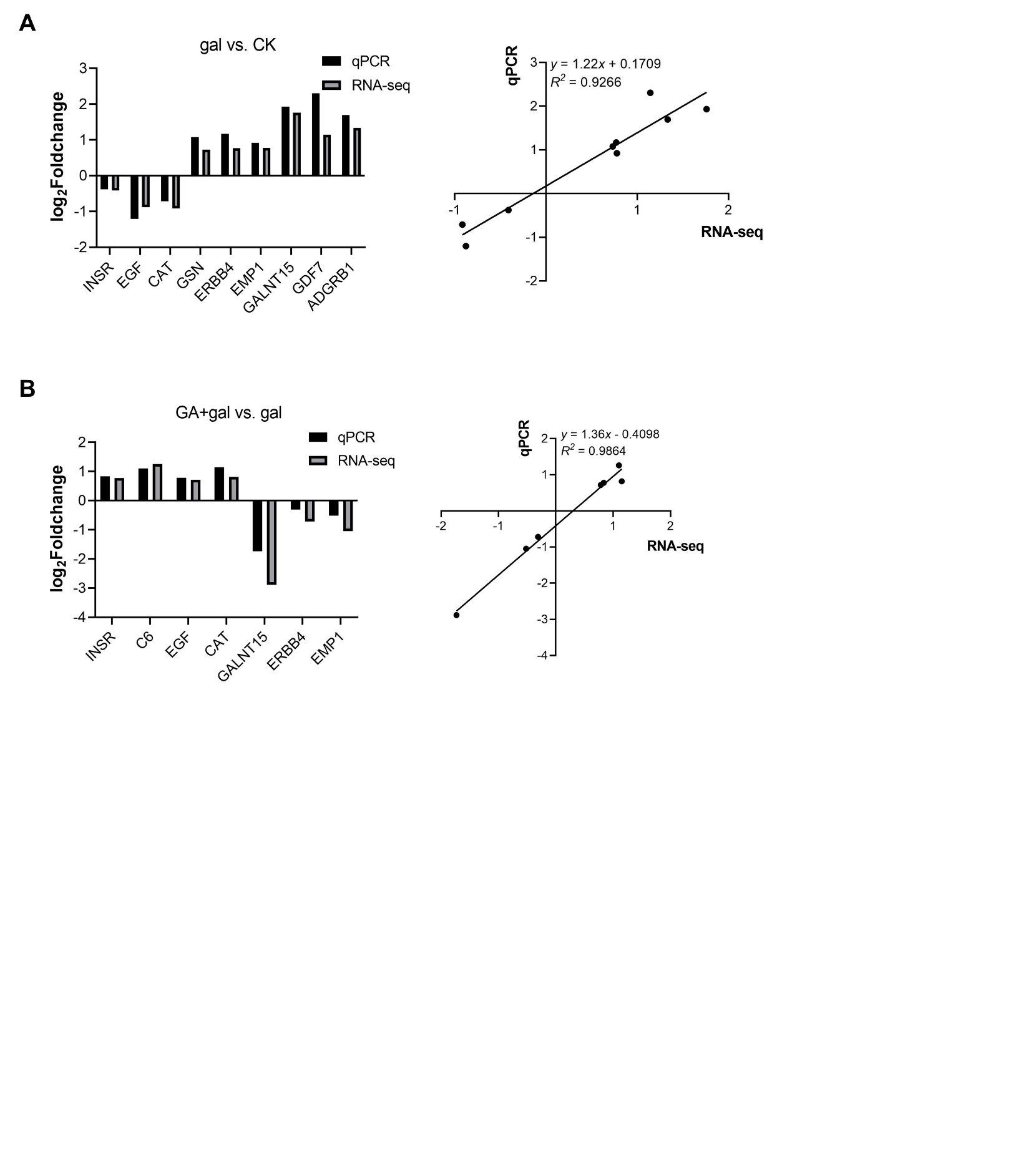


Figure S3 Validation of DEGs. Differential gene expression was concurrently assessed using RT-qPCR and RNA-seq, with log2 fold changes presented in correlation plots for (A) gal vs. CK and (B) GA + gal vs. gal (n = 3).
